# MicroRNAs as Biomarkers for Diagnosis, Prognosis and Theranostics in Prostate Cancer

**DOI:** 10.3390/ijms17030421

**Published:** 2016-03-22

**Authors:** Gloria Bertoli, Claudia Cava, Isabella Castiglioni

**Affiliations:** Institute of Molecular Bioimaging and Physiology, National Research Council (IBFM-CNR), Segrate 20090, Segrate (Mi), Italy; claudia.cava@ibfm.cnr.it (C.C.); isabella.castiglioni@ibfm.cnr.it (I.C.)

**Keywords:** prostate cancer, microRNAs, diagnosis, prognosis, therapy, theranostics, biomarkers

## Abstract

Prostate cancer (PC) includes several phenotypes, from indolent to highly aggressive cancer. Actual diagnostic and prognostic tools have several limitations, and there is a need for new biomarkers to stratify patients and assign them optimal therapies by taking into account potential genetic and epigenetic differences. MicroRNAs (miRNAs) are small sequences of non-coding RNA regulating specific genes involved in the onset and development of PC. Stable miRNAs have been found in biofluids, such as serum and plasma; thus, the measurement of PC-associated miRNAs is emerging as a non-invasive tool for PC detection and monitoring. In this study, we conduct an in-depth literature review focusing on miRNAs that may contribute to the diagnosis and prognosis of PC. The role of miRNAs as a potential theranostic tool in PC is discussed. Using a meta-analysis approach, we found a group of 29 miRNAs with diagnostic properties and a group of seven miRNAs with prognostic properties, which were found already expressed in both biofluids and PC tissues. We tested the two miRNA groups on The Cancer Genome Atlas dataset of PC tissue samples with a machine-learning approach. Our results suggest that these 29 miRNAs should be considered as potential panel of biomarkers for the diagnosis of PC, both as *in vivo* non-invasive test and *ex vivo* confirmation test.

## 1. Introduction

Prostate cancer (PC) is the most commonly diagnosed male tissue cancer and the second leading cause of tumor death in the United States [1]. It includes several phenotypes, from indolent to highly aggressive phenotypes, despite the fact that it tends to grow more slowly than other solid cancers.

Circulating prostate-specific antigen (PSA) is currently the most common non-invasive biomarker used to detect PC. However, the blood levels of PSA are often elevated in men with benign conditions (e.g., prostatitis, urinary tract infection, or benign prostatic hyperplasia). A low detection rate for PC has been associated with PSA in the so-called grey zone (4–10 ng/mL), and no advantage of PSA screening is recognized for healthy subjects [2].

The Gleason score, a histological grading method determined by needle biopsy [2] is affected by an inherent degree of subjectivity. Undergrading of the tumour from the a higher Gleason score needle biopsy compared to the matched whole gland is a common problem (42%) [3]. Furthermore, large aggressive lesions may not be properly characterized by either the Gleason score needle biopsy (when randomly performed within the tumour volume) or by PSA level [4]. The PC antigen 3 (PCA-3), has been suggested as a urine biomarker for the non-invasive early detection of PC, but it is not sufficient to avoid a second biopsy for all patients [5].

Although the number of patients diagnosed with PC is increasing, only one third die of castration-resistant metastatic disease [1]. Active surveillance is thus an option in the management of PC to limit complications of radiotherapy or radical prostatectomy. If patients with low Gleason scores (≤6) are assigned to an active surveillance program, and patients with high Gleason scores (≥8) are assigned to a definitive therapy, one problem is the management of patients with Gleason scores of 7 and high PSA levels [1], a patient group that is often over-treated without effectiveness. Recently, a classification system for PC using prognostic groups that better reflect the true biological aggressiveness of PC has been proposed. The new prognostic grading specifies the following 5 prognostic groups, based on Gleason score: prognostic group I (P1) (Gleason score ≤ 6); prognostic group II (P2) (Gleason score 3 + 4 = 7); prognostic group III (P3) (Gleason score 4 + 3 = 7); group IV (P4) (Gleason score 4 + 4 = 8); prognostic group V (P5) (Gleason score 9–10) [6,7]. This system has been validated on a large population [8] and demonstrated a 5-year progression-free survival rates of 94.6%, 82.7%, 65.1%, 63.1%, and 34.5%, respectively, for the five groups as determined by biopsy.

Although imaging has a minor role in PC diagnosis and staging, limits of PSA, Gleason score and PCA-3 have pushed the development of new imaging methods for PC such as multiparametric magnetic resonance imaging (mpMRI) [4] for staging the extent of disease and characterizing the radiological phenotype of the tumour [9]. However, the role of mpMRI for assigning a single patient to an optimal therapy is under study.

MicroRNAs (miRNAs) are short (18–22 nucleotides) non-coding RNA sequences with a wide regulatory function on molecular signalling pathways in the cell. They are appealing targets for screening, diagnosis, prognosis, monitoring tumour progression, biomarker discovery and assisting in identifying the correct treatment for patients. As they regulate the expression of specific target genes, including tumour suppressors and oncogenes, their dysregulation is involved in the alteration of several molecular processes in tissues leading to the complexity and heterogeneity of several cancers. Studies of miRNA isolation and characterization are based on either tissue samples or biofluids since miRNAs are extremely stable in serum, plasma and urine, making them particularly appealing for non-invasive tests.

The first studies on PC-altered miRNAs began in 2007–2008 with the purpose to identify miRNA profiles with diagnostic, prognostic and predictive abilities [10,11]. Since then, several miRNA profiles have been proposed for PC [12,13].

The aim of this paper is to review current scientific knowledge on miRNAs that may contribute to the diagnosis and prognosis of PC. A discussion on the potential role of miRNAs for new PC therapy development is also presented.

The review is based on an extensive literature search for research papers evaluating miRNAs in PC. Research was independently conducted in this search by two independent authors (Gloria Bertoli and Claudia Cava) by both PubMed and Google Scholar databases using the following key words (with both extended names and abbreviations): “miRNA AND prostate cancer”. In addition, reference lists of identified papers were hand searched to obtained additional articles. The search was concluded on December 2015. Finally, nine papers were added during the revision process of the manuscript updating the search to January 2016. Papers were considered for inclusion only if (a) they provided full text in the English language in a peer-reviewed journal; (b) were published from July 2007 to January 2016; and (c) included single miRNA or miRNA profiles with potential diagnostic, prognostic, and theranostic application to PC. Review articles included in the considered papers were searched for additional citations, however, books and book chapters were excluded. All publications were screened on their relevance for this paper. The final resulting papers were considered eligible for the systematic review.

## 2. miRNA Biogenesis Altered in Prostate Cancer (PC)

**Figure 1:**
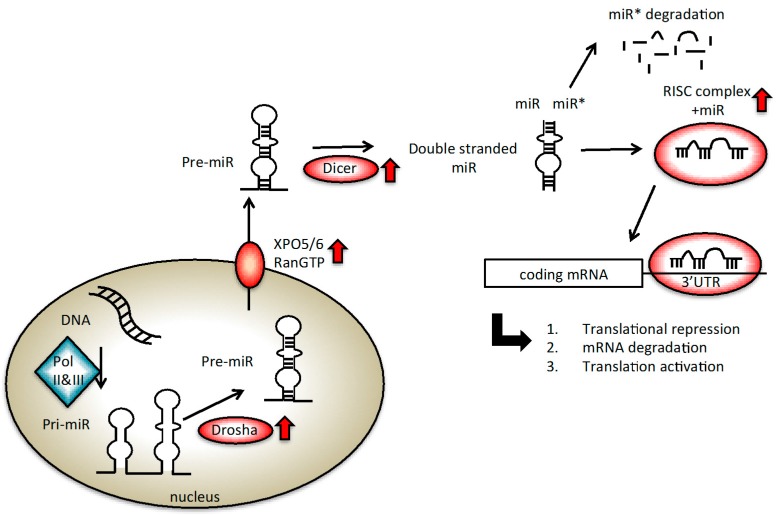
miRNA biogenesis. Red arrows indicate steps altered in prostate cancer (PC). The standard steps of the biogenesis are indicated with the black arrows. miRNAs are transcribed by RNA Polymerase (Pol) II or III to generate initially a sequence, called pri-miRNA, of approximately 200 bp. Afterwards, Drosha RNase III cleaves the pri-miRNA into a 100 nt sequence, called pre-miRNA, which is then exported out of the nucleus by exportin 5/6 (XPO5/6) and RanGTP. Another RNAse III, Dicer, generates a double stranded miRNA containing a miRNA/miRNA* duplex. The mature miRNA is loaded into the RNA-induced silencing complex (RISC) complex. The complementarity of the sequence between the miRNA and the mRNA leads either to translational repression if matching is imperfect, mRNA degradation if the seed is completely recognized, or translational activation, which occurs less frequently.

miRNAs are encoded by a large precursor RNA called pri-miR by RNA polymerase II or III (RNA Pol II or III) (Figure 1). It is then converted into mature miRNA of 19–25 nucleotides by Drosha, the nuclear RNAse III enzyme able to generate the pre-miR, and by Dicer, the cytosolic RNAse III enzyme fundamental for the long RNA duplex generation [14,15] (Figure 1). These two enzymes and their cofactors, *i.e.*, DiGeorge Syndrome Critical Region Gene 8 (DGCR8)/Pasha, Trans-Activation-Responsive RNA-Binding Protein (TRBP), Argonaute 2 (EIF2C2/Ago2), are essential for the miRNA maturation process. Indeed, one of the two strands is incorporated into a complex, containing also Ago2 protein, to form the RNA-induced silencing complex (RISC). Through the interaction with the RISC complex, miRNA regulates protein-coding gene expression by either repressing translation or cleaving RNA transcripts in a sequence complementarity-based procedure.

Changes in the level of expression of each of the protein members of the miRNA processing machinery can alter cell function, thus, inducing tumoural transformation (Figure 1, steps highlighted in red). In 2008, Ambs and colleagues found significantly higher expression of Double-Stranded RNA-Specific Endoribonuclease (Dicer) (+1.6-fold) and DGCR8 (+1.2-fold) in PC *vs.* normal tissues [16,17]. Moreover, several components of the RISC complex (*i.e.*, Dicer, EIF2C2) were found to be increased in PCs with high Gleason scores (7–9) than in PCs with lower Gleason scores [18]. In the same work, a higher expression of exportin 6 (XPO6), one of the proteins responsible for exporting miRNA from the nucleus to the cytosol, was described in PC [16].

At the end of their biogenesis, some of the deregulated miRNAs can be released, either passively or actively, from tumour cells into the surrounding environment. Extracellular miRNAs are found to be associated with proteins (*i.e.*, Ago2) and lipoproteins, packaged within cellular structures (*i.e.*, exosomes, microvescicles, or apoptotic bodies), and as cell-free molecules [19,20]. Several hypotheses have been proposed to justify the presence of extracellular miRNA in body fluids. A possibility is that miRNAs are actively secreted from a cell [21,22]. Other evidences suggest that miRNA release occurs as a passive mechanism, due to intracellular abundance of miRNA or passive release from broken and apoptotic cells [20,23]. Regardless of these two conflicting hypotheses, extracellular miRNAs are known to have a role in the communication between the tumoural cells and the environment [23,24,25].

## 3. miRNAs as Biomarkers of PC

### 3.1. Diagnostic miRNAs

#### 3.1.1. Intracellular Diagnostic miRNAs

The first attempts to find a miRNA profile of human PC was performed on PC cell lines [11,26]. Real time-PCR (RT-PCR) analysis identified that *miR-20*, *miR-21*, *miR-99a*, *miR-141*, *miR-182*, *miR-198* and *let-7a* were overexpressed, while *miR-145* and *miR-155* were downregulated in PC cell lines [26]. *miR-99a*, *miR-200c* and *miR-375* were also present in another diagnostic signature [27]. In 2010, two other diagnostic miRNA profiles on PC tissues were proposed. Szczyrba and colleagues found 33 dysregulated miRNAs in PC *vs.* normal (N) tissues using a deep sequencing technique [28]. Among them, the most significantly altered miRNAs with a diagnostic ability were *miR-148a*, *miR-200c*, *miR-375* (all upregulated) and *miR-143*, *miR-145* and *miR-223* (all downregulated). In 2010, Schaefer and colleagues identified a miRNA profile by microarray analysis of 24 PC (Gleason score ≥ 5) *vs.* 24 normal specimens tissue [29]. They found 15 dysregulated miRNAs in PC samples (10 downregulated and 5 upregulated). Of these miRNAs, six miRNAs were shown to have the best diagnostic potential: *miR-96*, *miR-182*, *miR-375* (all upregulated in PC) and *miR-149*, *miR-181b* and *miR-205* (all downregulated in PC) [29]. To gain further insight into the properties of miRNAs as diagnostic biomarkers, the same group published a miRNA profile in 2012 using a general-purpose commercial miRNA microarray on 20 PC tissues *vs.* 20 matched normal samples [30]. Their analysis revealed a group of 72 differentially expressed miRNAs; this list was reduced to 25 miRNAs after a validation process. A Receiver Operating Characteristic (ROC) analysis revealed that *miR-145*, *miR-200c* and *miR-375* were able to correctly distinguish PC and non-tumour samples [30].

In 2012, a miRNA expression microarray dataset of PC cancer tissues from the Gene Expression Omnibus (GEO) database was used to identify differentially expressed miRNAs in PC tissue as compared with normal tissue. Sixteen miRNAs were found to be differentially expressed [31]. Among them, *miR-145*, *miR-221* and *miR-222* were downregulated, in common with another study [11] that also reported downregulation of *miR-21*, *miR-30c* and *let-7c* in malignant tissues compared to normal ones. *miR-93*, *miR-145*, *miR-182* and *miR-221* have also been found by a separate study [16] while *miR-145* has been included in the miRNA profile analysed by a another study [10]. Other studies have reported on *miR-182*, *miR-375*, *miR-145*, *miR-221* and *miR-222* [29,30], with *miR-145*, *miR-182* and *miR-375* having diagnostic properties [30]. More recently, the analysis of 12 miRNAs was conducted on 75 PC and 27 benign samples, of either tissue or blood, by RT-PCR approach. Considered miRNAs were *miR-16*, *miR-21*, *miR-34a*, *miR-141*, *miR-143*, *miR-145*, *miR-155*, *miR-125b*, *miR-221*, *miR-375*, *miR-425* and *let-7a*. Four of the miRNAs were significantly altered in PC *vs.* N tissue samples with *miR-141*, *miR-145* and *miR-155* being upregulated, while *let-7a* was downregulated. The diagnostic power of this four miRNA-based signature was proven with a sensitivity of 97% and Area Under the Curve (AUC) of 0.783 [32]. In 2014, Casanova-salas analysed miRNAs differentially expressed in 50 PC *vs.* 50 normal tissues by microarray [33]. The authors found five upregulated miRNAs (miR-34*, *miR-34c*, *miR-187*, *miR-221*, *miR-224*) and a downregulated one (*miR-182*) able to distinguish PC from normal tissues. Given the fold-change expression of *miR-182* and *miR-187*, the authors focused on these two miRNAs for further validation analyses, which confirmed the altered expression of these miRNAs on a large retrospective group of 273 PC patients [33].

**Figure 2:**
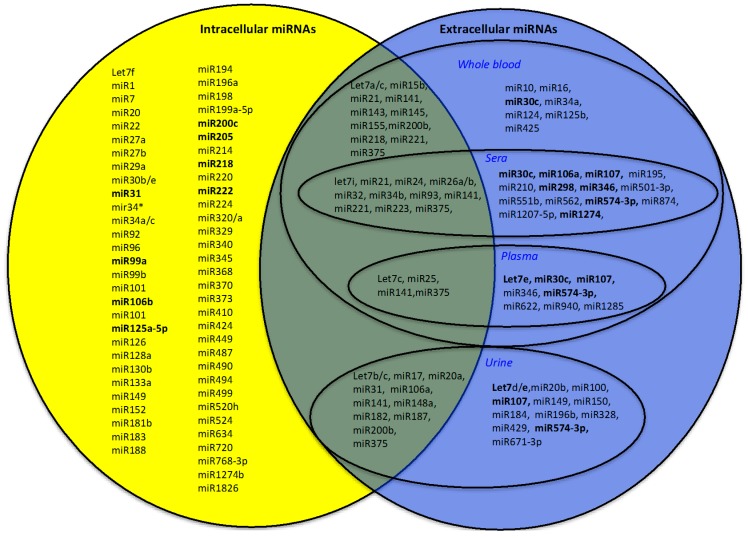
Venn diagram: extracellular and intracellular diagnostic miRNAs in PC. In bold: miRNAs altered in more than one signature. At the intersection of the sets: miRNAs in common among extracellular and intracellular diagnostic miRNAs.

All the described miRNAs are presented in Table 1. Although many discrepancies are emerging among PC miRNA expression profiles [34,35], this may be due to cellular heterogeneity and multifocal nature of PC, as well as to the different technical characteristics of the detection systems utilized [28]. Analysing all the proposed profiles of Table 1, we observed eight intracellular miRNAs (*let-7a*, *miR-21*, *miR-99a*, *miR-141*, *miR-145*, *miR-200c*, *miR-221*, *miR-375*) altered in PC tissues in common among multiple signatures (Table 1; Figure 2, miRNAs indicated in bold in the set of intracellular miRNAs).

#### 3.1.2. Extracellular Diagnostic miRNAs

For extracellular miRNA analysis, we considered both miRNAs found in circulating biofluids, such as whole blood, plasma and serum, and those secreted in saliva and urine. We excluded from our research those miRNAs found in cerebrospinal fluid (CSF) or seminal fluid because of the invasive nature of the collection of CSF and of the lack of publications on seminal fluid. Few studies considered the expression profile of miRNAs expressed in biofluids of PC patients. High variability in the proposed extracellular miRNA profiles is likely derived from differences in the techniques used for RNA extraction, types of samples used (whole blood, urine, serum and plasma), extraction methods and the use of different endogenous controls. Moreover, evidences suggest that ribonuclease (RNAse) activity is present in the serum of PC patients, making miRNA extraction more difficult [32].

One approach to studying miRNAs in PC is to analyse the miRNA profile of exosomes coming from tumoural PC3 cells and the non-cancerous prostate cell line, named RWPE-1. Hessvik and colleagues identified 29 miRNAs specifically present in PC-derived exosomes by microarray and validated them by RT-PCR [36]. Among them, *miR-4258* and *miR-711* are more highly expressed in exosomes of PC3 than in parental cells, while *miR-221*, *miR-193a-3p*, *miR-30e*, *miR-1297*, *miR-129-3p* and *miR-21-3p* are expressed at lower levels in PC3 exosomes than in parental cells [36].

The first results on extracellular miRNAs have been obtained from studying by RT-PCR analysis the miRNA profile of plasma and serum in a xenografted mouse model of PC (generated by inoculating the 22 Rv1 prostate human cancer cell line in non-obese diabetic/severe combined immunodeficiency, NOD/SCID, immunocompromised mice) [37]. This study demonstrated that *miR-141* serum level can distinguish samples coming from PC from healthy controls [37]. In another study, by microarray and RT-PCR, miRNA profiles were analysed in serum of a PC mouse model, and *miR-141*, *miR-298*, *miR-346* and *miR-375* were proposed as biomarkers of disease [38]. *miR-141* and *miR-375* were also confirmed in the serum and plasma of PC patients, respectively, by Lodes and colleagues [39] and by Brase and colleagues [42], both in a microarray-based approach. Lodes and colleagues also identified a group of 15 miRNAs (*miR-16*, *miR-92a*, *miR-103*, *miR-107*, *miR-197*, *miR-34b*, *miR*-*328*, *miR-485-3p*, *miR-486-5p*, *miR-92b*, *miR-574-3p*, *miR-636*, *miR-640*, *miR-766*, *miR-885-5p*) up-regulated in serum from PC patients compared to healthy donor sera (*n* = 6 and *n* = 8, respectively) [39].

In 2010, Moltzahn and colleagues collected the serum samples of 29 PC patients and nine healthy controls by multiplex RT-PCR [40]. In their study, they found 10 miRNAs altered in PC but only two miRNAs with diagnostic capability: *miR-106a* and *miR-1274*. The analysis of serum from 25 patients with PC *vs.* 17 with benign hyperplastic form revealed that *let-7c*, *let-7e*, *miR-30c*, *miR-662* and *miR-1285* are able to distinguish the two sample set, with the first three downregulated and the other two upregulated in PC samples [46]. Although the up-regulation of *miR-141* in extracellular fluids has previously been reported [38] and confirmed in the plasma of metastatic PC patients [41] by RT-PCR, some issues came from the study of Egidi [50] in which the author suggested that the observed upregulation of *miR-21* and *miR-141* in serum of patients subjected to radical prostatectomy is possibly due to the inflammatory process as opposed to the presence of PC, as these two miRNAs are involved in inflammatory processes.

In 2011, Moltzahn *et al.* analysed by multiplex RT-PCR method 36 PC *vs.* 12 normal patient sera and found 10 statistically different expressed miRNAs with diagnostic properties, including *miR-20b*, *miR-24*, *miR-26b*, *miR-30c*, *miR-93*, *miR-106a*, *miR-223*, *miR-874*, *miR-1207-5p*, and *miR-1274a* [40]. Some of these miRNAs have been already found in both tissue miRNA profiling [28] and extracellular miRNA profiling (*i.e.*, *miR-223*, *miR-24*) [41].

Also, Brase and colleagues found that extracellular *miR-375* and *miR-141* were highly expressed in patients with advanced PC, and their upregulation correlated with a high Gleason score or with the lymph-node positivity [42]. Mahn *et al.* profiled four different PC-associated miRNAs: *miR-26a*, *miR-32*, *miR-195* and *let-7i* by RT-PCR [43]. In particular, they found that *miR-26a*, *miR-195* and *let-7i* are increased in the serum of PC patients *vs.* the serum of benign hyperplasia patients, with this signature able to distinguish the two groups [43].

A 21-miRNA signature was proposed with significantly altered expression in urine of a small group of patients with PC [45]. In detail, a group of 19 miRNAs (*let-7b/c/d/e/g*, *miR-17*, *miR-20a/b*, *miR-31*, *miR-100*, *miR-106a*, *miR-148a*, *miR-149*, *miR-184*, *miR-196b*, *miR-200b*, *miR-429*, *miR-574-3p* and *miR-671-3p*) were significantly upregulated; *miR-150* and *miR-328* were down-regulated.

Most recently, Bryant and colleagues performed a high-throughput analysis of a wide range of miRNAs in serum, plasma and urinary samples. They found that plasmatic *miR-107* and *miR-574-3p* were highly expressed in the urine of patients with PC with respect to healthy subjects. This signature demonstrated the greatest ability to separate the two populations [44].

Haldrup and colleagues, by studying miRNA profiling of 13 benign prostatic hyperplasia (BPH) control patients and 31 PC patients, developed a miRNA profile able to identify 84% of all PC patients: *miR-210*, *miR-375*, *miR-501-3p*, *miR-551b* and *miR-562* [45]. The analyses of urine samples of 92 PC patients confirmed the deregulation of *miR-182* and *miR-187*, already found in PC tissues [33]. The authors proposed a prediction model for PC diagnosis, including several known PC biomarkers (serum PSA, urine PCA-3) and *miR-187*. They demonstrated that this predictive model achieved good diagnostic performance and greater accuracy than PSA alone [33].

Analysing the whole blood samples of 75 PC *vs.* 27 normal samples, Kelly *et al.* found a 12 miRNA profile, suggesting a potential diagnostic role for *miR-141*, *miR-145*, *miR-155* (all upregulated) and *let-7a* (downregulated) [32]. In 2015, Kachakova and colleagues obtained better diagnostic performance by combining the expression levels of *let-7c*, *miR-30c*, *miR-141*, *miR-375*, obtained by RT-PCR, with PSA among 16 BPH and 11 young asymptomatic men [48].

All the profiles describing extracellular miRNAs with a diagnostic role are presented in Table 1. We observed eight extracellular miRNAs (*let-7a/c/i*, *miR-21*, *miR-106a*, *miR-141*, *miR-375*, *miR-574-3p*) in common in multiple signatures altered in PC fluids (Table 1; Figure 2, miRNAs indicated in bold in the set of extracellular miRNAs).

Looking for miRNAs in common between extracellular and intracellular signatures, we identified a group of 29 miRNAs (*let-7a/b/c/i*, *miR-15b*, *miR-17*, *miR-20a*, *miR-21*, *miR-24*, *miR-25*, *miR-26a/b*, *miR-31*, *miR-32*, *miR-34b*, *miR-93*, *miR-106a*, *miR-141*, *miR-143*, *miR-145*, *miR-148a*, *miR-155*, *miR-182*, *miR-187*, *miR-200b*, *miR-218*, *miR-221*, *miR-223* and *miR-375*) (Figure 2). The Venn diagram in Figure 2 shows these miRNA in the central area shared between the area of the sets of intracellular and extracellular miRNAs. For some of these miRNAs, a functional mechanism in the PC onset has been proposed: the let7 family, for instance, and *miR-145* have a role in the control of proliferation, either via CCND2 and c-Myc mRNAs’ regulation, respectively [51,52]. *miR-15* and *miR-17* are able to cooperate with the microenvironment, enhancing cell survival, tumour expansion and invasiveness [53,54]. *miR-21* and *miR-221* are responsible for the control of cellular proliferation in several cancers [55,56]; *miR-25* is involved in the control of invasion via modulation of integrin expression [57].

### 3.2. Prognostic miRNAs

#### 3.2.1. Intracellular Prognostic miRNAs

Before 2010, few studies had been published on the prognostic role of miRNAs in PC and most were performed on a small number of tissue samples (approximately 10) and with largely divergent results [10,11,16,58,59].

In 2010, Schaefer and colleagues analysed 24 tissue pairs (PC *vs.* normal tissue), identifying, by miRNA-microarray approach, a miRNA profile with prognostic potential [29]. Fifteen differentially expressed miRNAs were identified with 10 downregulated and five upregulated. Of those miRNAs, *miR-31*, *miR-96*, *miR-125b*, *miR-205* and *miR-222* were found to be correlated with Gleason score. The authors also confirmed that *miR-96*, combined with Gleason scores, is a prognostic multimodal biomarker in PC [29].

In 2011, Leite and colleagues compared the miRNA profiles of 18 PC patients with Gleason scores between 8–10 (non-metastatic and metastatic) with those obtained from six patients with benign prostate hyperplasia (BPH) by RT-PCR [60]. The authors found six miRNAs (*let-7c*, *miR-100*, *miR-145*, *miR-146*, *miR-199a* and *miR-218*) up-regulated in the non-metastatic samples and *miR-145* was downregulated in the metastatic samples.

In 2012, Martens-Uzunova *et al.* identified a group of 54 miRNAs able to distinguish good prognosis from poor prognosis in 114 PC patient tissues by deep sequencing-based method [61]. In particular, they validated *miR-1*, *miR-143*, *miR-145*, *miR-205*, *miR-210*, *miR-222* and *miR-451*.

In 2013, Tsuchiyama and colleagues studied miRNA profile with respect to the standard three classes of risks [62]. Tissues of patients in the different classes were analysed by RT-PCR for the expression of *miR-31-5p*, *miR-34c-5p*, *miR-96-5p*, *miR-182-5p*, *miR-183-5p*, *miR-205-5p*, *miR-221-3p* and *miR-222-3p*. The authors found that the relative expression of *miR-31-5p*, *miR-182-5p* and *miR-205-5p* were differentially expressed between intermediate and high-risk patients.

Lichner and colleagues analysed miRNA deregulated in PC and found an association between high Gleason scores in PC and *miR-29*, *miR-34a* and *miR-141*, which are involved in the control of the extracellular matrix, cytoskeleton proteins and androgen receptor pathways using TaqMan-based miRNA array [63].

In the same year, Schubert and colleagues analysed the miRNA profiles, obtained by miRNA microarray, in a high-risk PC patient group [64]. They identified seven miRNAs (*let-7a/b/c*, *miR-146b*, *miR-181b*, *miR-361* and *miR-515-3p/5p*) able to stratify the patients into the three different risk groups. In particular, *let-7b* and *let-7c* were associated with high risk PC.

In 2014, a study on *miR-182* and *miR-187* demonstrated that *miR-182* expression strongly correlated with the prognostic factors of biochemical progression-free survival (BPFS) and progression-free survival (PFS), as well as between *miR-182* and Gleason score with patients with Gleason scores <7 and low *miR-182* expression considered at lower risk of progression compared with those with higher Gleason score (>7) and *miR-182* overexpression [33].

Recently, a tool named miQ with prognostic performance has been proposed, which consists of a combination of *miR-96-5p*, *miR-145-5p*, *miR-183-5p* and *miR-221-5p*, with the prognostic ability to predict tumor aggressiveness, metastatic status and overall survival in a Dutch cohort [65]. In particular, *miR-96* has been proposed to follow the postoperative outcome in more aggressive disease stages and *miR-96* expression was correlated with recurrence after surgery [66].

**Figure 3:**
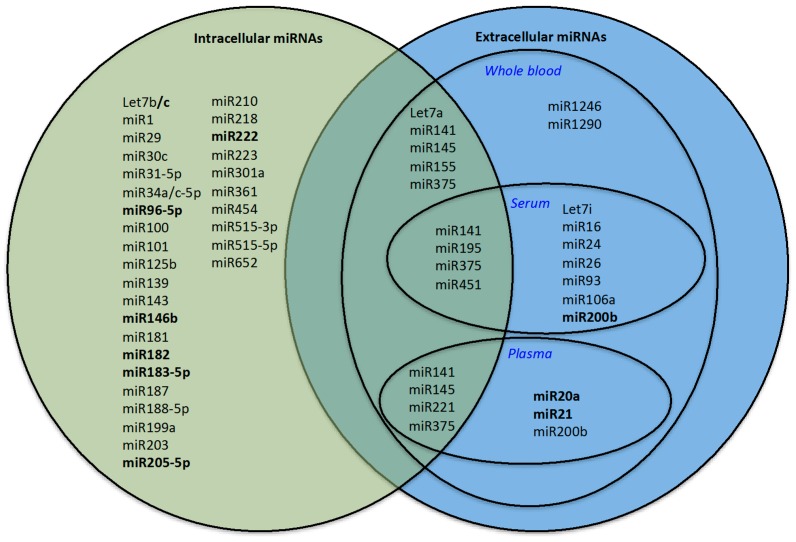
Venn diagram: Extracellular and intracellular prognostic miRNAs. In bold: miRNAs altered in more than one signature. At the intersection of the sets: miRNAs in common among extracellular and intracellular prognostic miRNAs.

Recently, the upregulation of *let-7a* and *miR-141* was found to be correlated with a higher risk group of patients. In addition, after retropubic-prostatectomy, the collected blood showed a reduction in *miR-141* expression to a level comparable to those of patients with benign histological findings [32]. *miR-375* was proposed with *miR-182* as prognostic biomarkers in [67], which suggested that higher *miR-375* expression is associated with higher Gleason score patients and more advanced pathological stages, as well as with regional lymph node metastases. Several other studies exist in which single miRNAs, identified by RT-PCR approach, have been proposed as prognostic tools for PC, *i.e.*, *miR-182* [68], *miR-30c* and *miR-203* [69], *miR-101* [70], *miR-195* [71], *miR-150* [72], *miR-188-5p* [73]. In particular, *miR-139*, *miR-223*, *miR-301a*, *miR-454*, and *miR-652* were found to be predictors of metastasis by next generation sequencing (NGS) approach [74]. All the profiles describing intracellular miRNAs with a prognostic role are presented in Table 2. We observed eight intracellular miRNAs (*let-7c*, *miR-96-5p*, *miR-145*, *miR-146*, *miR-182*, *miR-183-5p*, *miR-205-5p* and *miR-222*) in common in multiple signatures altered in PC tissues (Table 2; Figure 3, miRNAs indicated in bold in the set of intracellular miRNAs).

#### 3.2.2. Extracellular Prognostic miRNAs

Moltzahn *et al.* analysed sera before radical prostatectomy from 29 PC patients belonging to three classes of risks (The University of California, San Francisco (UCSF) Cancer of the Prostate Risk Assessment score, CAPRA score) by RT-PCR [48]. Analyses identified that *miR-24*, *miR-93*, *miR-106a* and *miR-451* were associated with the CAPRA score, thus proving their prognostic power. In the study by Brase, the analysis of 69 differentially expressed mRNAs between primary PC serum samples (*n* = 14) *vs.* metastatic serum samples (*n* = 7) demonstrated that *miR-141*, *miR-200b* and *miR-375* were increased in higher tumour stage and Gleason score patients [42]. Analysis of serum of PC patients (37 with clinically localized PC who underwent radical prostatectomy, 18 with BPH, 8 with metastatic PC and 20 healthy volunteers) demonstrated that three miRNAs (*miR-16*, *miR-195* and *miR-26a*) were significantly correlated with surgical margin positivity, while two miRNAs (*miR-195* and *let-7i*) were significantly correlated with the Gleason score [54].

Shen and colleagues investigated by RT-PCR the plasma miRNAs of 82 PC patients to associate miRNA expression with the development and progression of PC [76]. Results suggested that *miR-20a*, *miR-21*, *miR-145* and *miR-221* could predict the aggressiveness of PC. In particular, *miR-20a* is more expressed in stage 3 PC patients compared to stage 2 or below, and *miR-20a* and *miR-21* are highly expressed in patients with high risk.

A recent study identified 16 deregulated miRNAs in the plasma of men with localized (*n* = 55) *vs.* metastatic (*n* = 11) PC [44]. In this study, the authors demonstrated the association of *miR-141* and *miR-375* with metastatic disease. A prospective multi-centre study on serum samples of 133 patients described a significant increase of *miR-141* in patients with higher Gleason scores by RT-PCR analysis [75].

Kelly *et al.* found the upregulation of *let-7a* and *miR-141* in the blood and tissue samples of PC patients correlated to the higher risk group. In particular, the authors demonstrated that the expression levels of *miR-141*, as measured in the blood, indicating oncogenic characteristics, returned to normal levels on day 10 post-prostatectomy [32].

Furthermore, embedded extracellular exosomal *miR-375* and *miR-1290* have been significantly associated with poor prognosis in PC [69].

All the profiles describing extracellular miRNAs with a prognostic role are presented in Table 2.

Searching for miRNAs in common between extracellular and intracellular signatures, we identified a group of seven miRNAs (*let-7a*, *miR-141*, *miR-145*, *miR-195*, *miR-221*, *miR-375* and *miR-451*). The Venn diagram in Figure 3 shows these miRNAs in the central area shared between the area of intracellular and extracellular miRNA sets. For some of these miRNAs, a functional mechanism in PC onset has been proposed; *miR-141*, *miR-195*, and *miR-375* have been associated with PC metastasis [28,77,78] while *miR-145* and *miR-221* may control proliferation of PC cells [10,79].

### 3.3. In-Silico Assessment of miRNA Signatures Found with Meta-Analysis

In this section, we assessed the two miRNA signatures (with diagnostic and prognostic roles, respectively) found in common among intracellular and extracellular miRNAs. These biomarkers have already been validated in experimental studies in body fluids and in PC tissues and, thus, could be more easily proposed for clinical validation as a unique panel of miRNAs for a two-level clinical test (*in vivo*, pre- biopsy and *ex vivo*, as confirmation test, post-biopsy). However, stable intracellular expression of the other miRNA species found by our meta-analysis does not preclude them from being excellent extracellular biomarkers and the extracellular miRNAs found with the same approach may well be stably expressed inside the cell.

In order to validate the diagnostic and prognostic miRNA signatures, we used a support vector machine (SVM) model based on a rapid miner workflow (RMA-WF) [80]. The PC dataset was downloaded from The Cancer Genome Atlas (TGCA) database (of tissue-based expression levels of miRNAs), the most comprehensive repository of human cancer molecular and clinical data, using an R Bioconductor package, TGCAbiolinks [81].

We tested both the 29-miRNA diagnostic signature and the 7-miRNA prognostic signature. In order to avoid unbalanced samples, we considered the following samples: (i) for testing the diagnostic signature, we considered 52 PC and 52 normal samples; (ii) for testing the prognostic signature, we considered 138 samples from P1 + P2 samples, 138 from P3 + P4 samples and 138 samples from P5 samples. P1 and P2 prognostic groups had Gleason Scores of 6 and 3 + 4, respectively, and demonstrated five-year rates of biochemical progression-free survival (FS) of 94.6% and 82.7%, respectively. P3 + P4 was the prognostic group with Gleason Scores of 4 + 3 = 7 and 8, respectively, and demonstrated 5-year rates of FS of 65.1% and 63.1%, respectively. P5 was the prognostic group with Gleason Scores of 9–10 with a demonstrated FS of 34.5% [6,7].

Accuracy and AUC of SVM classification were estimated by cross-validation. We applied a *k*-fold cross-validation method, with *k* = 10. Confidence intervals (CI) were defined at the 95% level. We optimized accuracy using some SVM feasible learning parameters: kernel γ and kernel C ∈ {0…5} step 30; kernel type = RADIAL, DOT, ANOVA (see Rapid Miner documentation [80]).

As indicated in the Table 3, the accuracy and AUC of the 29-miRNA in classifying PC patients with respect to Normal samples is high, suggesting that this miRNA profile can be a reliable tool in detecting PC patients *vs.* healthy people in the same independent data set.

The seven miRNAs with prognostic ability demonstrated poor performances on the considered independent TCGA data sets (Table 4). In fact, as indicated in Table 4, the accuracy and AUC of the 7-miRNA signature in PC prognosis is poor for P1 + P2 *vs.* P5 (71.38% and 74.7%, respectively), for P3 + P4 *vs.* P5 (61.59% and 61.6%, respectively) and for P1 + P2 *vs.* P3 + P4 (65.26% and 66.8%, respectively). Better understanding of their role as prognostic biomarkers is needed with respect to the new risk classification for PC patients [6,7].

### 3.4. Pitfalls and Caveats of miRNA-Based Diagnostic and Prognostic Tools

In order to apply the best approach for the future development of miRNA-based non-invasive body-fluid based tests for PC diagnosis, some factors need to be considered. One of the challenges is the standardization of the procedures for extracellular miRNA purification. As miRNA profiling is a multistep process (including blood collection, plasma or serum isolation, RNA purification, RNA quantification and quality control and RNA profiling), each of these steps could be affected by methodological aspects and possible pitfalls [82]. Therefore, a great effort is needed to establish common and standardized procedures for the best practice. Another challenge is the normalization of data in analysing extracellular miRNAs [83]. The concentrations of some non-coding small nuclear RNAs (*i.e.*, small nuclear ribonucleoprotein U6 or Small Nucleolar RNA, C/D Box 44 SNORD44) have been used for sample-to-sample normalization. In the case of urine, creatinine has been proposed as a normalizer of concentration. It is likely to consider the use of other metabolites or proteins to normalize plasma and urine miRNA measurements [83]. A third crucial challenge in order to have a diagnostic tool using miRNA profiles is the calculation of an optimal unique cut-off value, able to discriminate between PC patients and normal subjects.

As there is little agreement on the reported prognostic individual miRNAs and miRNA signatures among studies from intracellular and extracellular samples, it is difficult to collect a significant group of miRNA with prognostic ability.

In order to be most effective in uncovering the best prognostic markers, some concerns should be consider in the better choice of the patients enrolled in a study design. The ideal clinical study to identify prognostic miRNAs should enrol a large cohort of PC patients divided in different groups following the prognostic classification proposed in [6,7]: P1 including Gleason score ≤ 6; P2 including Gleason score 3 + 4; P3 including Gleason score 4 + 3; P4 including Gleason score = 8; P5 including Gleason score = 9–10. This approach would overcome the problem of PC heterogeneity with respect to prognosis. A study design optimal for obtaining miRNAs predicting the outcome of treatment could be a prospective study on two groups of PC patients, the first one including patients with recurrence after radical prostatectomy, the second one consisting into responding patients. Another possible approach could consider healthy members of a family with PC history and compared them with healthy members of a family without PC history, who should be followed during their life, collecting biofluids for miRNA extraction and analysis. The comparison of their miRNA profile could help in the discovery of new PC prognostic tools. Despite the identification of multiple candidate molecules, very few tissue biomarkers have been validated across different patient groups, and fewer have been adopted as tools for clinical use [84]. Moreover, these putative biomarkers have been isolated without a random sampling scheme from a well-defined population, or on very small patient cohort, with limited clinical information and incomplete follow-up, thus making them useless to be used as prognostic biomarkers [84].

## 4. Therapeutic miRNAs

Understanding the role of miRNAs in PC development could lead to the generation of novel miRNA-based therapies. The main approaches for therapeutic miRNA use would include: (i) targeting the 3′UTR of the oncogenes controlled by PC-downregulated miRNA, using miRNA mimic oligonucleotides or constructs; (ii) restoring normal intracellular levels of upregulated miRNAs, by using antago-oligonucleotide (antago-miR) or synthetic constructs that deplete the cells of specific miRNAs (called miRNA sponge) and (iii) combining miRNA-based tools with existing anticancer drugs.

Several attempts have been made using PC animal models in which the modulation of single miRNAs affected tumour proliferation and development. Intra-tumoural administration of *miR-199a-3p* mimic oligonucleotides in a PC xenograft mouse model reduced the expression of the Aurora kinase A (AurkA) oncogene, whose expression correlated with a malignant PC phenotype [85]. Recently, it has been demonstrated that reduced *miR-15*/*16* cluster expression and upregulation of *miR-21* expression are critical events in the onset of PC metastasis. In fact, the downregulation of *miR-15* and *miR-16* in RAS-transformed PC cell lines, injected in the sub-renal capsules (in order to enter the bloodstream), enhanced the local invasion and metastatic activity into the bones of immunocompromised mice. RAS activation promotes the expression of *miR-21*, helping the development of bone metastasis [86]. *miR-16*-based therapy has been also proposed to treat bone metastasis in a PC mouse model, demonstrating a role for this miRNA in the regulation of cell-cycle genes [87].

A mouse model of PC was also used to demonstrate that the treatment of tumoural cells with a cellular-permeable peptide containing *miR-145* increased cellular sensitivity to radiotherapy. The *in vitro* study then demonstrated that *miR-145* overexpression reduced the efficiency of the repair mechanism of radiation-induced DNA double strand breaks in the cells, leading to apoptosis of PC cells [88].

Several attempts have been made to demonstrate a role of *miR-375* in the control of cellular proliferation. Moreover, evidence suggests that *miR-375* is one of the most significantly downregulated miRNAs in cancer and may have a role as a tumour suppressor [89]. Cholesterol-conjugated *miR-375* has been used in a mouse model of hepatocellular carcinoma (HCC) and demonstrated the stability of the molecule and the effect of *miR-375* on cell growth inhibition [89,90]. The same inhibitory effect of *miR-375* overexpression has been described *in vitro* and *in vivo* in gastric cancer [91,92] but no research was found on *miR-375*-based therapeutic treatments for PC. This is perhaps due to a hypothesized dual role of *miR-375* as either an onco-miR or a tumour suppressor miRNA, depending on the cellular context, in androgen receptor regulation or the tumour model [67].

Over-expression of *miR-143* and *miR-145* by retrovirus-based transfection decreased the capability of PC-3 cell lines of migration and invasion *in vitro*, and tumour development and bone invasion *in vivo* [93]. In addition, xenograft mouse models of PC obtained by PC-3 cell injection confirmed the capability of both let-7a and *miR-200b* to inhibit prostate tumour development *in vivo* [94,95]. A clear role of *miR-221/222* on cell proliferation and growth control has been demonstrated *in vitro* [79] and *in vivo* in PC xenografted mouse models [96].

*miR-34*, whose loss is reported in several cancer types, comprises three family members [97]. *miR-34c-5p* is among the prognostic PC intracellular miRNAs, and its homologue, *miR-34a*, is one of the possible prognostic miRNAs for high Gleason score PC. *miR-34a* is able to control several processes, such as the cell cycle, apoptosis, metastasis and cancer stemness. This is the reason why *miR-34* mimic oligonucleotides have been developed and successfully used in PC xenografted mice as a miRNA-based therapeutic tool [98]. *miR-34a* has also been used in combination with conventional therapies, such as camptothecin [99] and paclitaxel [100].

All the considered therapeutic miRNAs, *i.e.*, the miRNAs demonstrated to potentially influence the PC progression or sensitivity to chemo/radio-therapy, are also present in the group of the diagnostic miRNAs described before. Therefore, *let-7a*, *miR-15/16*, *miR-21*, *miR-141*, *miR-143*, *miR-145*, *miR-199a-3p*, *miR-200b*, *miR-221/222* could be proposed as potential *theranostic* tools in PC. One of the prognostic intracellular miRNAs associated with high Gleason score PC, *miR-34*, has already been considered as a possible miRNA-based therapeutic tool. The systemic delivery of *miR-34* mimic oligonucleotides has been successful in blocking PC growth in xenografted mouse models [98].

## 5. Hallmarks of PC as Possible miRNA-Based Therapies

**Figure 4:**
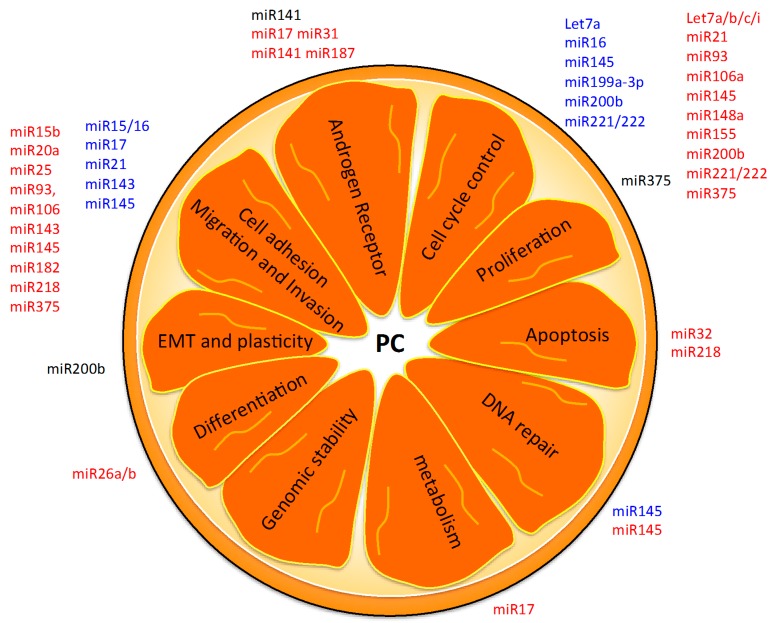
Possible actions of miRNA-based therapeutic strategies. Here we report miRNAs (regulators of PC altered functions) as possible targets for the development of new therapeutic tools. Diagnostic (red), prognostic (black) and theranostic (blue) miRNAs are indicated. EMT = epithelial-to-mesenchymal transition; PC = prostate cancer.

Considering the altered functions that guide PC development, we analysed the main functions controlled by the described diagnostic, prognostic and theranostic proposed miRNAs (Figure 4). Three are the main hallmarks controlled by diagnostic miRNAs. For example, some miRNAs are involved in proliferation control (*i.e.*, *let-7* family member, *miR-16*, *miR-21*, *miR-145*, *miR-182*, *miR-199a-3p* and *miR-221*) [101]; *miR-15/16*, *miR-17*, *miR-143* are involved in tumour proliferation and communication with the microenvironment [53,54,100]; another miRNA (*miR-145*) is responsible for the control of DNA repair mechanisms after radiation therapy and deciding whether a cell should undergo apoptosis [88].

The analysis of altered miRNA expression in PC revealed that the proposed group of prognostic miRNAs (*miR-141*, *miR-200b* and *miR-375*) are involved in the control of androgen receptor expression [79], membrane plasticity of cells [95] and DNA repair modulation [67], respectively (Figure 4). Theranostic miRNAs (*let-7a*, *miR-15/16*, *miR-21*, *miR-141*, *miR-143*, *miR-145*, *miR-199a-3p*, *miR-200b* and *miR-221/222*) are mainly devoted to the control of the cell cycle, growth and proliferation [52], as well as modulation of cell adhesion molecules to regulate migration and invasion capability of the cells [53,54,100] (Figure 4). One prognostic miRNA, *miR-34a*, has been used for the development of *miR-34* mimic oligonucleotides able to block PC cell growth *in vivo* [102,103].

## 6. Conclusions

In this paper, we reviewed the literature focusing on miRNAs with a role in the diagnosis and prognosis of PC, and discussed their potential theranostic role in PC. By a meta-analysis approach, we found a group of 29 miRNAs with diagnostic properties (*let-7a/b/c/i*, *miR-15b*, *miR-17*, *miR-20a*, *miR-21*, *miR-24*, *miR-25*, *miR-26a/b*, *miR-31*, *miR-32*, *miR-34b*, *miR-93*, *miR-106a*, *miR-141*, *miR-143*, *miR-145*, *miR-148a*, *miR-155*, *miR-182*, *miR-187*, *miR-200b*, *miR-218*, *miR-221*, *miR-223* and *miR-375*) and a group of 7 miRNAs with prognostic properties expressed in both PC tissues and biofluids. We tested the two miRNA groups on a TCGA dataset of PC tissue samples with a machine learning approach. Our results suggest that the profile of 29 miRNAs should be considered as potential diagnostic tools in PC as a non-invasive blood test.

## Figures and Tables

**Table 1:** miRNAs with a role in the diagnosis of PC. Extracellular and intracellular miRNAs are indicated. PC = prostate cancer, N = normal, BPH = benign prostatic hyperplasia, SF = seminal fluid, RT-PCR = real time-PCR.

Intracellular miRNAs	Cohort and Tissue Sample	Discovery Method	Reference
*let-7a, miR-20, miR-21, miR-99a, miR-141, miR-182, miR-198; miR-145, miR-155*	5 PC *vs.* 4 N cell line	miRNA array and RT-PCR	[11,26]
*let-7i, miR-25, miR-26a, miR-31, miR-32, miR-34b, miR-92, miR-93, miR-99b, miR-106b, miR-125a, miR-181a, miR-182, miR-188, miR-194, miR-196a, miR-200c, miR-370, miR-375, miR-425, miR-449; let-7b, miR-1, miR-7, miR-34a, miR-126, miR-128a, miR-133a, miR-145, miR-205, miR-218, miR-220, miR-221, miR-329, miR-340, miR-345, miR-410, miR-487, miR-490, miR-494, miR-499, miR-520h*	60 PC *vs.* 16 N tissues	miRNA array and RT-PCR	[16]
*miR-148a, miR-200c, miR-375, let-7a, let-7c, let-7f, miR-15b, miR-20a, miR-21, miR-25, miR-26b, miR-30b, miR-106a/b, miR-126-3p, miR-218; miR-143, miR-145; miR-223, miR-22, miR-24, miR-27a, miR-27b, miR-29a, miR-30e, miR-101, miR-125a-5p, miR-125b, miR-152, miR-199a-5p, miR-221, miR-320, miR-424*	10 PC *vs.* 10 N tissues	Small RNA cloning and deep sequencing	[28]
*miR-96, miR-182, miR-375, miR-130b, miR-182-3p, miR-183, miR-524, miR-634; miR-149, miR-181b, miR-205, miR-31, miR-125b, miR-145, miR-184, miR-205, miR-221, miR-222, miR-373, miR-368*	24 PC *vs.* 24 N tissues	miRNA array and RT-PCR	[29]
*miR-200b/c, miR-375, let-7a, miR-17, miR-20a/b, miR-21, miR-93, miR-101, miR-106a/b, miR-141, miR-182, miR-720, miR-768-3p, miR-1274b, miR-1826; miR-145, miR-136-3p, miR-214, miR-221, miR-222,miR-302d-3p, miR-378-3p*	20 PC *vs.* 20 N tissues	miRNA array and RT-PCR	[30]
*miR-17, miR-20a/b, miR-93, miR-101, miR-106a, miR-141, miR-145, miR-182, miR-214, miR-221, miR-222, miR-320a, miR-375, miR-720, miR-768-3p*	20 PC *vs.* 20 N tissues	miRNA array analysis	[31]
*miR-141, miR-145 and miR-155; let-7a*	75 PC *vs.* 27 benign samples, blood and tissues	RT-PCR	[32]
miR-34*, miR-34c, miR-221, miR-224, miR-182; miR-187	50 PC *vs.* 50 N tissues	miRNA array	[33]
*Extracellular miRNAs*			
*miR-7, miR-10a-3p, miR-146b-p, miR-155, miR-181a-2-3p, miR-326, miR-484, miR-485-3p, miR-489, miR-551a, miR-585, miR-601, miR-620, miR-622, miR-659, miR-676-3p, miR-874, miR-943, miR-1252, miR-1253, miR-1273e, miR-1298, miR-1470, miR-1915, miR-3620, miR-3663-5p, miR-3690, miR-4292, miR-4305*	PC3 cell lines and derived exosomes	miRNA array and RT-PCR	[36]
*miR-141*	xenografted mouse model; sera	RT-PCR	[37]
*miR-141, miR-298, miR-346, miR-375*	PC adenocarcinoma mouse model; sera	miRNA array and RT-PCR	[38]
*miR-16, miR-92a/b, miR-103, miR-107,miR-197, miR-34b, miR-328, miR-485-3p, miR-486-5p, miR-574-3p, miR-636, miR-640, miR-766, miR-885-5p*	6 PC *vs.* 8 N; sera	miRNA array	[39]
*miR-20b, miR-24, miR-26b, miR-30c, miR-93, miR-106a, miR-223, miR-874, miR-1207-5p, miR-1274a*	29 PC *vs.* 9 N sera	Multiplex RT-PCR	[40]
*miR-21, miR-30a, miR-99a, miR-141, miR-200b/c, miR-221, miR-298, miR-346, miR-375*	25 PC *vs.* 25 N; sera and SF	RT-PCR	[27,38,41]
*miR-375, miR-141*	10 PC metastatic *vs.* 59 localized PC *vs.* 48 high risk *vs.* 23 intermediate risk tumour; sera	miRNA array	[42]
*miR-93, miR-106a, miR-874, miR-1207-5p, miR-1274a; miR-24, miR-26b, miR-30c, miR-223*	36 PC *vs.* 12 N; sera	RT-PCR	[41]
*let--7i, miR-26a, miR-32, miR-195*	8 PC *vs.* 18 BPH *vs.* 20 N sera	RT-PCR	[43]
*miR-107, miR-141, miR-375, miR-574-3p*	78 PC *vs.* 28 N of sera, plasma, urine	RT–PCR microarray	[44]
*miR-141, miR-210, miR-375, miR-501-3p, miR-551b, miR-562*	31 PC *vs.* 13 BPH sera	RT–PCR	[45]
*miR-346, miR-622, miR-940, miR-1285; let-7e, let-7c, miR-25, miR-30c*	105 PC *vs.* 61BPH *vs.* 54 N plasma	miRNA array	[46]
*miR-15b, miR-200b, miR-218*	40 localised PC *vs.* N; whole blood	miRNA array	[47]
*miR-16, miR-21, miR-34a, miR-125b, miR-141, miR-143, miR-145, miR-155, miR-221, miR-375, miR-425; let-7a*	102 from high Gleason score PC *vs.* N; whole blood	RT-PCR	[32]
*miR-15b, let-7c, miR-30c, miR-141, miR-375*	11 PC *vs.* 16 BPH; whole blood	RT-PCR	[48]
*let-7b/c/d/e, miR-17, miR-20a/b, miR-31, miR-100, miR-106a, miR-148a, miR-149, miR-184, miR-196b, miR-200b, miR-429, miR-574-3p, miR-671-3p, miR-150, miR-328*	Urine	RT-PCR	[49]

**Table 2:** miRNA selection with prognostic ability. Extracellular and intracellular prognostic miRNAs are indicated. NGS = next generation sequencing; PC = prostate cancer; N = normal sample.

Intracellular miRNAs	Cohort and Tissue Samples	Discovery Method	Reference
*miR-31, miR-96, miR-125b, miR-205, miR-222*	24 PC *vs.* 24 N; tissue	miRNA array	[29]
*let-7c, miR-100, miR-145, miR-146, miR-199a* and *miR-218*	18 PC *vs.* 6 BPH; tissue	RT-PCR	[60]
*miR-1, miR-143, miR-145, miR-205, miR-210, miR-222, miR-451*	4 PC *vs.* 4 N; tissue	Deep sequencing	[61]
*miR-31-5p, miR-34c-5p, miR-96-5p, miR-182-5p, miR-183-5p, miR-205-5p, miR-221-3p* and *miR-222-3p*	Gleason pattern 2 (*n* = 22), 4 (*n* = 35) 5 (*n* = 12); tissue	RT-PCR	[62]
*miR-29, miR-34a, miR-141*	High Gleason score PC (*n* = 45); tissue	miRNA array	[63]
*let-7a/c, miR-146b, miR-181b, miR-361, miR-515-3p/5p*	Different PC risk groups (*n* = 98); tissue	miRNA array	[64]
*miR-182, miR-187*	Gleason ≤ 7 (*n* = 302) or >7 (*n* = 46)	miRNA array	[33]
*miR-96-5p, miR-145-5p, miR-183-5p, miR-221-5p;* definition of miQ index	*n* = 25 PC *vs. n* = 25 N tissues	RT-PCR	[65]
*miR-182, miR-375*	*n* = 119 PC patients	miRNA array	[67]
*miR-301a, miR-652, miR-454, miR-223* and *miR-139*	*n* = 515 PC patients (metastatic *vs.* non-metastatic)	NGS	[74]
**Single prognostic miRNA**			
*miR-182*	*n* = 147 PC patients	RT-PCR	[68]
*miR-30c, miR-203*	*n* = 44 PC patients	RT-PCR	[69]
*miR-101*	*n* = 16 localized PC, *n* = 26 metastatic PC	RT-PCR	[70]
*miR-195*	*n* = 225 PC patients	RT-PCR	[71]
*miR-150*	*n* = 167 PC patients	RT-PCR	[72]
*miR-188-5p*	*n* = 180 PC patients	RT-PCR	[73]
**Extracellular miRNAs**			
*miR-24, miR-93, miR-106a, miR-451*	different risk PC (*n* = 29) (CAPRA score); sera	Multiplex RT-PCR	[40]
*miR-141, miR-200b* and *miR-375*	14 primary or 7 metastatic PC men; sera	miRNA array	[42]
*miR-195* and *let-7i*	37 localized PC, 18 BPH, 8 metastatic PC and 20 N; sera	RT-PCR	[43]
*miR-141* is increased in higher Gleason score patients	from 133 patients; sera	RT–PCR	[75]
*miR-21* and *miR-145* in patients with intermediate- or high-risk *miR-20a* and *miR-21* in high-risk *miR-20a, miR-21, miR-145* and *miR-221* in high- vs. low-risk PC men	low-, intermediate- and high-risk patients (82 patients); plasma	RT–PCR	[76]
*miR-141, miR-200b* and *miR-375*	55 localized PC patients *vs.* 11 metastatic PC; plasma	RT–PCR microarray	[44]
*let-7a, miR-141, miR-145 and miR-155*	75 PC and 27 N Blood and tissues	RT-PCR	[32]
*miR-1246, miR-1290, miR-375*	100 late-stage PC patients; blood	RT-PCR	[69]

**Table 3:** Accuracy and Area Under the Curve (AUC) for the 29 miRNA diagnostic signatures. CI = confidence interval; PC = prostate cancer; N = normal sample

Classification	29 miRNA Diagnostic Signature	Accuracy	AUC
PC *vs.* N 52 *vs.* 52	*Let-7a/b/c/i, miR-15b, miR-17, miR-20a, miR-21, miR-24, miR-25, miR-26a/b, miR-31, miR-32, miR-34b, miR-93, miR-106a, miR-141, miR-143, miR-145, miR-148a, miR-155, miR-182, miR-187, miR-200b, miR-218, miR-221, miR-223, miR-375*	97.18% +/− 4.31% (CI 95%): 96.35–98.00	0.989 +/− 0.016 (CI 95%): 98.59–99.20

**Table 4:** Accuracy and Area Under the Curve (AUC) for the seven miRNA prognostic signatures. CI = confidence interval.

Classification	7 miRNA Prognostic Signature	Accuracy	AUC
P1 + P2 *vs.* P5 138 *vs.* 138	*let-7a, miR-141, miR-145, miR-195, miR-221, miR-375, miR-451*	71.38% (CI 95%): 70.79–71.96	74.7% (CI 95%): 73.28–76.11
P3 + P4 *vs.* P5 138 *vs.* 138	*let-7a, miR-141, miR-145, miR-195, miR-221, miR-375, miR-451*	61.59% (CI 95%): 60.71–62.46	61.6% (CI 95%): 60.24–62.95
P1 + P2 *vs.* P3 + P4 164 *vs.* 164	*let-7a, miR-141, miR-145, miR-195, miR-221, miR-375, miR-451*	65.26% (CI 95%): 64.51–66.00	66.8% (CI 95%): 66.10–67.49

## Abbreviations

PC: prostate cancer
miRs or miRNAs: microRNAs
N: normal tissue
T: tumor tissue

## References

[1] Johnson L.M., Choyke P.L., Figg W.D., Turkbey B. (2014). The role of MRI in prostate cancer active surveillance. BioMed Res. Int..

[2] Xu W., Zhou M. (2014). A concise update on prostate pathology. Ceskoslov. Patol..

[3] Humphrey P.A. (2004). Gleason grading and prognostic factors in carcinoma of the prostate. Mod. Pathol..

[4] Turkbey B., Brown A.M., Sankineni S., Wood B.J., Pinto P.A., Choyke P.L. (2015). Multiparametric prostate magnetic resonance imaging in the evaluation of prostate cancer. CA Cancer J. Clin..

[5] Vickers J., Thompson A., Collins G.S., Childs M., Hain R. (2007). Place and provision of palliative care for children with progressive cancer: A study by the Paediatric Oncology Nurses’ Forum/United Kingdom Children’s Cancer Study Group Palliative Care Working Group. J. Clin. Oncol..

[6] Carter H.B., Partin A.W., Walsh P.C., Trock B.J., Veltri R.W., Nelson W.G., Coffey D.S., Singer E.A., Epstein J.I. (2012). Gleason score 6 adenocarcinoma: Should it be labeled as cancer?. J. Clin. Oncol..

[7] Loeb S., Montorsi F., Catto J.W. (2015). Future-proofing gleason grading: What to call gleason 6 prostate cancer?. Eur. Urol..

[8] Pierorazio P.M., Walsh P.C., Partin A.W., Epstein J.I. (2013). Prognostic gleason grade grouping: Data based on the modified gleason scoring system. BJU Int..

[9] Moore C.M., Emberton M. (2015). Will the attributes of multiparametric MRI permit the creation of a new approach to therapy?. Curr. Opin. Urol..

[10] Ozen M., Creighton C.J., Ozdemir M., Ittmann M. (2008). Widespread deregulation of microRNA expression in human prostate cancer. Oncogene.

[11] Porkka K.P., Pfeiffer M.J., Waltering K.K., Vessella R.L., Tammela T.L., Visakorpi T. (2007). microRNA expression profiling in prostate cancer. Cancer Res..

[12] Rane J.K., Simms M.S., Maitland N.J. (2014). Re: Yves Allorya, Willemien Beukers, Ana Sagrera, *et al.* Telomerase reverse transcriptase promoter mutations in bladder cancer: High frequency across stages, detection in urine, and lack of association with outcome. Eur urol 2014;65:360–6: Telomerase expression and stem cells: Urologic epithelial perspective. Eur. Urol..

[13] Ren Q., Liang J., Wei J., Basturk O., Wang J., Daniels G., Gellert L.L., Li Y., Shen Y., Osman I. (2014). Epithelial and stromal expression of miRNAs during prostate cancer progression. Am. J. Transl. Res..

[14] Lin S., Gregory R.I. (2015). microRNA biogenesis pathways in cancer. Nat. Rev. Cancer.

[15] Bertoli G., Cava C., Castiglioni I. (2015). microRNAs: New biomarkers for diagnosis, prognosis, therapy prediction and therapeutic tools for breast cancer. Theranostics.

[16] Ambs S., Prueitt R.L., Yi M., Hudson R.S., Howe T.M., Petrocca F., Wallace T.A., Liu C.G., Volinia S., Calin G.A. (2008). Genomic profiling of microRNA and messenger RNA reveals deregulated microRNA expression in prostate cancer. Cancer Res..

[17] Belair C.D., Paikari A., Moltzahn F., Shenoy A., Yau C., Dall’Era M., Simko J., Benz C., Blelloch R. (2015). DGCR8 is essential for tumor progression following PTEN loss in the prostate. EMBO Rep..

[18] Chiosea S., Jelezcova E., Chandran U., Acquafondata M., McHale T., Sobol R.W., Dhir R. (2006). Up-regulation of dicer, a component of the microRNA machinery, in prostate adenocarcinoma. Am. J. Pathol..

[19] Hessvik N.P., Sandvig K., Llorente A. (2013). Exosomal miRNAs as biomarkers for prostate cancer. Front. Genet..

[20] Javidi M.A., Ahmadi A.H., Bakhshinejad B., Nouraee N., Babashah S., Sadeghizadeh M. (2014). Cell-free microRNAs as cancer biomarkers: The odyssey of miRNAs through body fluids. Med. Oncol..

[21] Pigati L., Yaddanapudi S.C., Iyengar R., Kim D.J., Hearn S.A., Danforth D., Hastings M.L., Duelli D.M. (2010). Selective release of microRNA species from normal and malignant mammary epithelial cells. PLoS ONE.

[22] Sita-Lumsden A., Dart D.A., Waxman J., Bevan C.L. (2013). Circulating microRNAs as potential new biomarkers for prostate cancer. Br. J. Cancer.

[23] Fong M.Y., Zhou W., Liu L., Alontaga A.Y., Chandra M., Ashby J., Chow A., O’Connor S.T., Li S., Chin A.R. (2015). Breast-cancer-secreted miR-122 reprograms glucose metabolism in premetastatic niche to promote metastasis. Nat. Cell Biol..

[24] Le M.T., Hamar P., Guo C., Basar E., Perdigao-Henriques R., Balaj L., Lieberman J. (2014). miR-200-containing extracellular vesicles promote breast cancer cell metastasis. J. Clin. Investig..

[25] Kosaka N., Iguchi H., Yoshioka Y., Hagiwara K., Takeshita F., Ochiya T. (2012). Competitive interactions of cancer cells and normal cells via secretory microRNAs. J. Biol. Chem..

[26] Roa W., Brunet B., Guo L., Amanie J., Fairchild A., Gabos Z., Nijjar T., Scrimger R., Yee D., Xing J. (2010). Identification of a new microRNA expression profile as a potential cancer screening tool. Clin. Investig. Med. Med. Clin. Exp..

[27] Selth L.A., Roberts M.J., Chow C.W., Marshall V.R., Doi S.A., Vincent A.D., Butler L.M., Lavin M.F., Tilley W.D., Gardiner R.A. (2014). Human seminal fluid as a source of prostate cancer-specific microRNA biomarkers. Endocr. Relat. Cancer.

[28] Szczyrba J., Loprich E., Wach S., Jung V., Unteregger G., Barth S., Grobholz R., Wieland W., Stohr R., Hartmann A. (2010). The microRNA profile of prostate carcinoma obtained by deep sequencing. Mol. Cancer Res..

[29] Schaefer A., Jung M., Mollenkopf H.J., Wagner I., Stephan C., Jentzmik F., Miller K., Lein M., Kristiansen G., Jung K. (2010). Diagnostic and prognostic implications of microRNA profiling in prostate carcinoma. Int. J. Cancer.

[30] Wach S., Nolte E., Szczyrba J., Stohr R., Hartmann A., Orntoft T., Dyrskjot L., Eltze E., Wieland W., Keck B. (2012). MicroRNA profiles of prostate carcinoma detected by multiplatform microRNA screening. Int. J. Cancer.

[31] Liu D.F., Wu J.T., Wang J.M., Liu Q.Z., Gao Z.L., Liu Y.X. (2012). MicroRNA expression profile analysis reveals diagnostic biomarker for human prostate cancer. Asian Pac. J. Cancer Prev..

[32] Kelly B.D., Miller N., Sweeney K.J., Durkan G.C., Rogers E., Walsh K., Kerin M.J. (2015). A circulating microRNA signature as a biomarker for prostate cancer in a high risk group. J. Clin. Med..

[33] Casanova-Salas I., Rubio-Briones J., Calatrava A., Mancarella C., Masia E., Casanova J., Fernandez-Serra A., Rubio L., Ramirez-Backhaus M., Arminan A. (2014). Identification of miR-187 and miR-182 as biomarkers of early diagnosis and prognosis in patients with prostate cancer treated with radical prostatectomy. J. Urol..

[34] Catto J.W., Shariat S.F. (2013). The changing face of renal cell carcinoma: The impact of systematic genetic sequencing on our understanding of this tumor′s biology. Eur. Urol..

[35] Coppola V., de Maria R., Bonci D. (2010). MicroRNAs and prostate cancer. Endocr. Relat. Cancer.

[36] Hessvik N.P., Phuyal S., Brech A., Sandvig K., Llorente A. (2012). Profiling of microRNAs in exosomes released from PC-3 prostate cancer cells. Biochim. Biophys. Acta.

[37] Mitchell P.S., Parkin R.K., Kroh E.M., Fritz B.R., Wyman S.K., Pogosova-Agadjanyan E.L., Peterson A., Noteboom J., O’Briant K.C., Allen A. (2008). Circulating microRNAs as stable blood-based markers for cancer detection. Proc. Natl. Acad. Sci. USA.

[38] Selth L.A., Townley S., Gillis J.L., Ochnik A.M., Murti K., Macfarlane R.J., Chi K.N., Marshall V.R., Tilley W.D., Butler L.M. (2012). Discovery of circulating microRNAs associated with human prostate cancer using a mouse model of disease. Int. J. Cancer.

[39] Lodes M.J., Caraballo M., Suciu D., Munro S., Kumar A., Anderson B. (2009). Detection of cancer with serum miRNAs on an oligonucleotide microarray. PLoS ONE.

[40] Moltzahn F., Olshen A.B., Baehner L., Peek A., Fong L., Stoppler H., Simko J., Hilton J.F., Carroll P., Blelloch R. (2011). Microfluidic-based multiplex qRT-PCR identifies diagnostic and prognostic microRNA signatures in the sera of prostate cancer patients. Cancer Res..

[41] Yaman Agaoglu F., Kovancilar M., Dizdar Y., Darendeliler E., Holdenrieder S., Dalay N., Gezer U. (2011). Investigation of miR-21, miR-141, and miR-221 in blood circulation of patients with prostate cancer. Tumour Biol..

[42] Brase J.C., Johannes M., Schlomm T., Falth M., Haese A., Steuber T., Beissbarth T., Kuner R., Sultmann H. (2011). Circulating miRNAs are correlated with tumor progression in prostate cancer. Int. J. Cancer.

[43] Mahn R., Heukamp L.C., Rogenhofer S., von Ruecker A., Muller S.C., Ellinger J. (2011). Circulating microRNAs (miRNA) in serum of patients with prostate cancer. Urology.

[44] Bryant R.J., Pawlowski T., Catto J.W., Marsden G., Vessella R.L., Rhees B., Kuslich C., Visakorpi T., Hamdy F.C. (2012). Changes in circulating microRNA levels associated with prostate cancer. Br. J. Cancer.

[45] Haldrup C., Kosaka N., Ochiya T., Borre M., Hoyer S., Orntoft T.F., Sorensen K.D. (2014). Profiling of circulating microRNAs for prostate cancer biomarker discovery. Drug Deliv. Transl. Res..

[46] Chen Z.H., Zhang G.L., Li H.R., Luo J.D., Li Z.X., Chen G.M., Yang J. (2012). A panel of five circulating microRNAs as potential biomarkers for prostate cancer. Prostate.

[47] Medina-Villaamil V., Martinez-Breijo S., Portela-Pereira P., Quindos-Varela M., Santamarina-Cainzos I., Anton-Aparicio L.M., Gomez-Veiga F. (2014). Circulating microRNAs in blood of patients with prostate cancer. Actas Urol. Esp..

[48] Kachakova D., Mitkova A., Popov E., Popov I., Vlahova A., Dikov T., Christova S., Mitev V., Slavov C., Kaneva R. (2015). Combinations of serum prostate-specific antigen and plasma expression levels of let-7c, miR-30c, miR-141, and miR-375 as potential better diagnostic biomarkers for prostate cancer. DNA Cell Biol..

[49] Ahumada-Tamayo S., Saavedra-Briones D., Cantellano-Orozco M., Salido-Guadarrama A., Rodríguez-Dorantes M., Urdiales-Ortiz A., Hernández-Castellanos V., Merayo-Chalico C., Sánchez-Turati G., Santana-Ríos Z. (2011). MicroRNA determination in urine for prostate cancer detection in Mexican patients at the hospital general “Dr. Manuelgea gonzález”. Rev. Mex. Urol..

[50] Egidi M.G., Cochetti G., Serva M.R., Guelfi G., Zampini D., Mechelli L., Mearini E. (2013). Circulating microRNAs and kallikreins before and after radical prostatectomy: Are they really prostate cancer markers?. BioMed Res. Int..

[51] Sachdeva M., Zhu S., Wu F., Wu H., Walia V., Kumar S., Elble R., Watabe K., Mo Y.Y. (2009). P53 represses c-Myc through induction of the tumor suppressor miR-145. Proc. Natl. Acad. Sci. USA.

[52] Wagner S., Ngezahayo A., Murua Escobar H., Nolte I. (2014). Role of miRNA let-7 and its major targets in prostate cancer. BioMed Res. Int..

[53] Musumeci M., Coppola V., Addario A., Patrizii M., Maugeri-Sacca M., Memeo L., Colarossi C., Francescangeli F., Biffoni M., Collura D. (2011). Control of tumor and microenvironment cross-talk by miR-15a and miR-16 in prostate cancer. Oncogene.

[54] Yang X., Du W.W., Li H., Liu F., Khorshidi A., Rutnam Z.J., Yang B.B. (2013). Both mature miR-17-5p and passenger strand miR-17-3p target TIMP3 and induce prostate tumor growth and invasion. Nucleic Acids Res..

[55] Park J.K., Lee E.J., Esau C., Schmittgen T.D. (2009). Antisense inhibition of microRNA-21 or -221 arrests cell cycle, induces apoptosis, and sensitizes the effects of gemcitabine in pancreatic adenocarcinoma. Pancreas.

[56] Xu L., Dai W.Q., Xu X.F., Wang F., He L., Guo C.Y. (2012). Effects of multiple-target anti-microRNA antisense oligodeoxyribonucleotides on proliferation and migration of gastric cancer cells. Asian Pac. J. Cancer Prev..

[57] Zoni E., van der Horst G., van de Merbel A.F., Chen L., Rane J.K., Pelger R.C., Collins A.T., Visakorpi T., Snaar-Jagalska B.E., Maitland N.J. (2015). miR-25 modulates invasiveness and dissemination of human prostate cancer cells via regulation of αv- and α6-integrin expression. Cancer Res..

[58] Tong A.W., Fulgham P., Jay C., Chen P., Khalil I., Liu S., Senzer N., Eklund A.C., Han J., Nemunaitis J. (2009). MicroRNA profile analysis of human prostate cancers. Cancer Gene Ther..

[59] Volinia S., Calin G.A., Liu C.G., Ambs S., Cimmino A., Petrocca F., Visone R., Iorio M., Roldo C., Ferracin M. (2006). A microRNA expression signature of human solid tumors defines cancer gene targets. Proc. Natl. Acad. Sci. USA.

[60] Leite K.R., Tomiyama A., Reis S.T., Sousa-Canavez J.M., Sanudo A., Dall’Oglio M.F., Camara-Lopes L.H., Srougi M. (2011). MicroRNA-100 expression is independently related to biochemical recurrence of prostate cancer. J. Urol..

[61] Martens-Uzunova E.S., Jalava S.E., Dits N.F., van Leenders G.J., Moller S., Trapman J., Bangma C.H., Litman T., Visakorpi T., Jenster G. (2012). Diagnostic and prognostic signatures from the small non-coding RNA transcriptome in prostate cancer. Oncogene.

[62] Tsuchiyama K., Ito H., Taga M., Naganuma S., Oshinoya Y., Nagano K., Yokoyama O., Itoh H. (2013). Expression of microRNAs associated with gleason grading system in prostate cancer: miR-182-5p is a useful marker for high grade prostate cancer. Prostate.

[63] Lichner Z., Fendler A., Saleh C., Nasser A.N., Boles D., Al-Haddad S., Kupchak P., Dharsee M., Nuin P.S., Evans K.R. (2013). MicroRNA signature helps distinguish early from late biochemical failure in prostate cancer. Clin. Chem..

[64] Schubert M., Spahn M., Kneitz S., Scholz C.J., Joniau S., Stroebel P., Riedmiller H., Kneitz B. (2013). Distinct microRNA expression profile in prostate cancer patients with early clinical failure and the impact of let-7 as prognostic marker in high-risk prostate cancer. PLoS ONE.

[65] Larne O., Martens-Uzunova E., Hagman Z., Edsjo A., Lippolis G., den Berg M.S., Bjartell A., Jenster G., Ceder Y. (2013). miQ—A novel microRNA based diagnostic and prognostic tool for prostate cancer. Int. J. Cancer..

[66] Haflidadottir B.S., Larne O., Martin M., Persson M., Edsjo A., Bjartell A., Ceder Y. (2013). Upregulation of miR-96 enhances cellular proliferation of prostate cancer cells through FOXO1. PLoS ONE.

[67] Costa-Pinheiro P., Montezuma D., Henrique R., Jeronimo C. (2015). Diagnostic and prognostic epigenetic biomarkers in cancer. Epigenomics.

[68] Wallis C.J., Gordanpour A., Bendavid J.S., Sugar L., Nam R.K., Seth A. (2015). miR-182 is associated with growth, migration and invasion in prostate cancer via suppression of FOXO1. J. Cancer.

[69] Huang Z., Zhang L., Yi X., Yu X. (2015). Diagnostic and prognostic values of tissue hsa-miR-30c and hsa-miR-203 in prostate carcinoma. Tumour Biol..

[70] Varambally S., Cao Q., Mani R.S., Shankar S., Wang X., Ateeq B., Laxman B., Cao X., Jing X., Ramnarayanan K. (2008). Genomic loss of microRNA-101 leads to overexpression of histone methyltransferase EZH2 in cancer. Science.

[71] Cai C., Chen Q.B., Han Z.D., Zhang Y.Q., He H.C., Chen J.H., Chen Y.R., Yang S.B., Wu Y.D., Zeng Y.R. (2015). miR-195 inhibits tumor progression by targeting RPS6KB1 in human prostate cancer. Clin. Cancer Res..

[72] Dezhong L., Xiaoyi Z., Xianlian L., Hongyan Z., Guohua Z., Bo S., Shenglei Z., Lian Z. (2015). miR-150 is a factor of survival in prostate cancer patients. J. BUON.

[73] Zhang H., Qi S., Zhang T., Wang A., Liu R., Guo J., Wang Y., Xu Y. (2015). miR-188-5p inhibits tumour growth and metastasis in prostate cancer by repressing LAPTM4B expression. Oncotarget.

[74] Nam R.K., Amemiya Y., Benatar T., Wallis C.J., Stojcic-Bendavid J., Bacopulos S., Sherman C., Sugar L., Naeim M., Yang W. (2015). Identification and validation of a five microRNA signature predictive of prostate cancer recurrence and metastasis: A cohort study. J. Cancer.

[75] Westermann A.M., Schmidt D., Holdenrieder S., Moritz R., Semjonow A., Schmidt M., Kristiansen G., Muller S.C., Ellinger J. (2014). Serum microRNAs as biomarkers in patients undergoing prostate biopsy: Results from a prospective multi-center study. Anticancer Res..

[76] Shen J., Hruby G.W., McKiernan J.M., Gurvich I., Lipsky M.J., Benson M.C., Santella R.M. (2012). Dysregulation of circulating microRNAs and prediction of aggressive prostate cancer. Prostate.

[77] Liu C., Guan H., Wang Y., Chen M., Xu B., Zhang L., Lu K., Tao T., Zhang X., Huang Y. (2015). miR-195 inhibits emt by targeting FGF2 in prostate cancer cells. PLoS ONE.

[78] Zhang H.L., Qin X.J., Cao D.L., Zhu Y., Yao X.D., Zhang S.L., Dai B., Ye D.W. (2013). An elevated serum miR-141 level in patients with bone-metastatic prostate cancer is correlated with more bone lesions. Asian J. Androl..

[79] Wang C., Tao W., Ni S., Chen Q., Zhao Z., Ma L., Fu Y., Jiao Z. (2015). Tumor-suppressive microRNA-145 induces growth arrest by targeting SENP1 in human prostate cancer cells. Cancer Sci..

[80] Mierswa I., Wurst M., Klinkenberg R., Scholz M., Euler T. Yale: Rapid prototyping for complex data mining tasks. Proceedings of the 12th ACM SIGKDD International Conference on Knowledge Discovery and Data Mining.

[81] Colaprico A., Silva T.C., Olsen C., Garofano L., Cava C., Garolini D., Sabedot T.S., Malta T.M., Pagnotta S.M., Castiglioni I. (2015). TCGabiolinks: An R/Bioconductor package for integrative analysis of TCGA data. Nucleic Acids Res..

[82] Moldovan L., Batte K.E., Trgovcich J., Wisler J., Marsh C.B., Piper M. (2014). Methodological challenges in utilizing miRNAs as circulating biomarkers. J. Cell. Mol. Med..

[83] Weber J.A., Baxter D.H., Zhang S., Huang D.Y., Huang K.H., Lee M.J., Galas D.J., Wang K. (2010). The microRNA spectrum in 12 body fluids. Clin. Chem..

[84] Hawley S., Fazli L., McKenney J.K., Simko J., Troyer D., Nicolas M., Newcomb L.F., Cowan J.E., Crouch L., Ferrari M. (2013). A model for the design and construction of a resource for the validation of prognostic prostate cancer biomarkers: The canary prostate cancer tissue microarray. Adv. Anat. Pathol..

[85] Qu Y., Huang X., Li Z., Liu J., Wu J., Chen D., Zhao F., Mu D. (2014). miR-199a-3p inhibits aurora kinase a and attenuates prostate cancer growth: New avenue for prostate cancer treatment. Am. J. Pathol..

[86] Bonci D., Coppola V., Patrizii M., Addario A., Cannistraci A., Francescangeli F., Pecci R., Muto G., Collura D., Bedini R. (2016). A microRNA code for prostate cancer metastasis. Oncogene.

[87] Takeshita F., Patrawala L., Osaki M., Takahashi R.U., Yamamoto Y., Kosaka N., Kawamata M., Kelnar K., Bader A.G., Brown D. (2010). Systemic delivery of synthetic microRNA-16 inhibits the growth of metastatic prostate tumors via downregulation of multiple cell-cycle genes. Mol. Ther..

[88] Gong P., Zhang T., He D., Hsieh J.T. (2015). MicroRNA-145 modulates tumor sensitivity to radiation in prostate cancer. Radiat. Res..

[89] Yan J.W., Lin J.S., He X.X. (2014). The emerging role of miR-375 in cancer. Int. J. Cancer..

[90] Chang Y., Yan W., He X., Zhang L., Li C., Huang H., Nace G., Geller D.A., Lin J., Tsung A. (2012). miR-375 inhibits autophagy and reduces viability of hepatocellular carcinoma cells under hypoxic conditions. Gastroenterology.

[91] Ding L., Xu Y., Zhang W., Deng Y., Si M., Du Y., Yao H., Liu X., Ke Y., Si J. (2010). miR-375 frequently downregulated in gastric cancer inhibits cell proliferation by targeting JAK2. Cell Res..

[92] Kong K.L., Kwong D.L., Chan T.H., Law S.Y., Chen L., Li Y., Qin Y.R., Guan X.Y. (2012). MicroRNA-375 inhibits tumour growth and metastasis in oesophageal squamous cell carcinoma through repressing insulin-like growth factor 1 receptor. Gut.

[93] Friedman R.C., Farh K.K., Burge C.B., Bartel D.P. (2009). Most mammalian mRNAs are conserved targets of microRNAs. Genome Res..

[94] Dong Q., Meng P., Wang T., Qin W., Qin W., Wang F., Yuan J., Chen Z., Yang A., Wang H. (2010). MicroRNA let-7a inhibits proliferation of human prostate cancer cells *in vitro* and *in vivo* by targeting E2f2 and CCND2. PLoS ONE.

[95] Williams L.V., Veliceasa D., Vinokour E., Volpert O.V. (2013). miR-200b inhibits prostate cancer EMT, growth and metastasis. PLoS ONE.

[96] Mercatelli N., Coppola V., Bonci D., Miele F., Costantini A., Guadagnoli M., Bonanno E., Muto G., Frajese G.V., De Maria R. (2008). The inhibition of the highly expressed miR-221 and miR-222 impairs the growth of prostate carcinoma xenografts in mice. PLoS ONE.

[97] Bader A.G. (2012). miR-34—A microRNA replacement therapy is headed to the clinic. Front. Genet..

[98] Liu C., Kelnar K., Liu B., Chen X., Calhoun-Davis T., Li H., Patrawala L., Yan H., Jeter C., Honorio S. (2011). The microRNA miR-34a inhibits prostate cancer stem cells and metastasis by directly repressing CD44. Nat. Med..

[99] Fujita Y., Kojima K., Hamada N., Ohhashi R., Akao Y., Nozawa Y., Deguchi T., Ito M. (2008). Effects of miR-34a on cell growth and chemoresistance in prostate cancer PC3 cells. Biochem. Biophys. Res. Commun..

[100] Kojima K., Fujita Y., Nozawa Y., Deguchi T., Ito M. (2010). miR-34a attenuates paclitaxel-resistance of hormone-refractory prostate cancer PC3 cells through direct and indirect mechanisms. Prostate.

[101] Sanchez C.A., Andahur E.I., Valenzuela R., Castellon E.A., Fulla J.A., Ramos C.G., Trivino J.C. (2016). Exosomes from bulk and stem cells from human prostate cancer have a differential microRNA content that contributes cooperatively over local and pre-metastatic niche. Oncotarget.

[102] Misso G., di Martino M.T., de Rosa G., Farooqi A.A., Lombardi A., Campani V., Zarone M.R., Gulla A., Tagliaferri P., Tassone P. (2014). miR-34: A new weapon against cancer?. Mol. Ther. Nucleic Acids.

[103] Mirna Therapeutics, Inc. microRNA Replacement Therapy. http://www.mirnarx.com/pipeline/mirna-MRX34.html.

